# Weight Loss and Physical Activity Reduces Perceived Physical Fatigability Among Older Adults With Obesity

**DOI:** 10.1002/oby.70298

**Published:** 2026-09-09

**Authors:** Reagan E. Garcia, Jason Fanning, Chaeryon Kang, Bonny Rockette‐Wagner, Christopher E. Kline, Barbara J. Nicklas, Nancy W. Glynn

**Affiliations:** ^1^ Department of Epidemiology University of Pittsburgh School of Public Health Pittsburgh Pennsylvania USA; ^2^ Department of Health and Exercise Sciences Wake Forest University Winston‐Salem North Carolina USA; ^3^ Department of Psychiatry University of Pittsburgh School of Medicine Pittsburgh Pennsylvania USA; ^4^ Department of Biostatistics and Health Data Science University of Pittsburgh School of Public Health Pittsburgh Pennsylvania USA; ^5^ Department of Health and Human Development University of Pittsburgh School of Education Pittsburgh Pennsylvania USA; ^6^ Department of Internal Medicine, Section on Gerontology and Geriatric Medicine Wake Forest University School of Medicine Winston‐Salem North Carolina USA

**Keywords:** aging, clinical trials, fatigue

## Abstract

**Objective:**

This study aimed to evaluate the impact of three lifestyle interventions on perceived physical fatigability.

**Methods:**

Concurrent with a dietary weight loss program (caloric deficit ~400 kcal/day, weekly dietitian support), older adults with obesity (*N* = 183) were randomized to three groups: aerobic exercise (150–200 min/week of moderate‐intensity treadmill walking; *n* = 60), daylong movement (improving daily activity distribution, progressively increase steps/day; *n* = 63), or combination (aerobic and daylong movement interventions; *n* = 60). Perceived physical fatigability (Pittsburgh Fatigability Scale [PFS] Physical score, range 0–50, and Rating of Perceived Exertion [RPE] after a 5‐min 0% grade treadmill walk, range 6–20; higher = greater fatigability) was measured at baseline, 6 months (post‐intensive intervention), and 18 months (end of maintenance period).

**Results:**

Participants (69.6 ± 4.3 years, 77% female, 74% White, 35.6 ± 3.8 kg/m^2^ BMI) had a PFS Physical score 20.6 ± 7.6 and RPE Fatigability 9.6 ± 1.9 at baseline. Clinically relevant improvements were observed in PFS score from baseline to 6 months (−4.4 points, *p* = 0.002) and were largely maintained at 18 months (−3.0 points, *p* = 0.03) after adjustment for baseline age, sex, education, intervention group, and time × intervention group (*p* = 0.83). RPE Fatigability did not improve (*p* > 0.05).

**Conclusions:**

Multiple physical activity strategies in conjunction with weight loss improved perceived physical fatigability over time among older adults with obesity.

**Trial Registration:**

ClinicalTrials.gov identifier: NCT02923674

## Study Importance

- What is already known?
  ○ Perceived physical fatigability, fatigue measured in the context of activity, is higher among older adults with obesity and is implicated in the age‐related disability pathway.
  ○ Evidence on whether a physical activity intervention can modify perceived physical fatigability is limited; no studies to date have examined this within the context of a weight loss program for older adults with obesity.
- What does this study add?
  ○ A weight loss and physical activity intervention led to clinically relevant improvements in perceived physical fatigability among older adults with obesity; the improvement was similar across three different physical activity intervention components, including an increase in low‐intensity movement throughout the day and structured moderate‐intensity walking.
  ○ Improvement in perceived physical fatigability was shown with 6 months of intensive weight loss and physical activity intervention and was largely maintained 1 year after intervention end among older adults with obesity.
- How might these results change the direction of research or the focus of clinical practice?
  ○ Our results show weight loss in combination with reaching 150 min/week of moderate‐vigorous physical activity or improving the distribution of daily activity can meaningfully reduce fatigability levels among older adults with obesity.
  ○ We demonstrated that perceived physical fatigability is modifiable and should be targeted to extend the healthspan of older adults with obesity.

## 1 Introduction

Despite overall increases in lifespan, the healthspan‐lifespan gap remains > 12 years among United States older adults, leading to lower quality of life and poor health during final years of older adulthood [1]. Identifying and intervening on early indicators of poor function are crucial to preserve health among older adults. Frequently experiencing higher levels of fatigue may be an early indicator of poor function among older adults as it significantly alters physical activity patterns over time and accelerates declines in overall aerobic capacity and physical function, thus furthering the age‐related capacity‐function decline [2]. This may be particularly true among older adults with obesity, as they tend to experience high fatigue levels and are at greater risk for disability [3, 4, 5].

However, fatigue, a non specific symptom, is difficult to measure accurately and most clinical measurements do not account for the bias introduced through self‐pacing behaviors, limiting the utility to compare between individuals or longitudinally [6, 7]. Perceived physical fatigability, fatigue measured in relation to anchored tasks of specific intensities and durations, is a more sensitive person‐reported assessment of fatigue as it standardizes the fatigue level to specific tasks [8, 9, 10]. Perceived physical fatigability (herein fatigability) is prevalent (20%–90%, higher among greater ages) among older adults, with a greater burden among older adults with obesity [11, 12]. It also serves as an early indicator of disability, as those with greater fatigability have 2 times higher odds of developing mobility disability and mortality than those with lower fatigability [13, 14]. Fatigability is also a marker of aerobic capacity and cardiorespiratory fitness [15, 16, 17, 18]. Thus, lowering fatigability among older adults is critical to extend population‐level healthspan.

Given the importance of physical activity for maintaining or improving aerobic capacity among older adults, physical activity may be an effective intervention to lower fatigability [19]. There are robust associations between physical activity and fatigability among older adults [20, 21, 22, 23, 24, 25]. Specifically, limited clinical trial evidence shows that exercise‐based interventions improve fatigability [26, 27, 28, 29]. However, these studies have been conducted in mostly younger cohorts of cancer survivors with small sample sizes [26, 27, 29]. Moreover, the interventions have not been well described, so it is difficult to extrapolate the minimum exercise dosage and intensity needed to lower fatigability for future translational interventions. It is also unknown how fatigability levels are maintained once the interventions taper. This information is needed to critically evaluate the long‐term success of lowering fatigability using physical activity. Thus, there is a strong need to rigorously examine and report the effectiveness of well‐defined lifestyle interventions on fatigability, particularly among older adults with obesity. While no study has examined the effects of a lifestyle intervention on fatigability among older adults with obesity, two studies found improved fatigue scores (i.e., vitality), with greater improvements among those who improved their activity and, notably, a twofold greater improvement among groups with exercise plus caloric restriction compared to exercise only [30, 31]. Given this, understanding the effects of weight loss interventions on fatigability among older adults with obesity is necessary as it may translate to meaningful improvement in quality of life, as well as decreased risk in later‐life disability.

The aim of this study was to compare the effectiveness of three weight loss plus physical activity interventions on fatigability among older adults with obesity using data from the Empowered with Movement to Prevent Weight Regain (EMPOWER) randomized clinical trial [32]. Given the robust associations between aerobic capacity, physical activity, and fatigability [18, 21], we hypothesized that the intervention groups that included an aerobic exercise component would lower fatigability more than the intervention group that included a daylong movement component.

## 2 Methods

The EMPOWER study was a parallel, three‐arm, single‐blinded randomized clinical trial designed to understand the effects of a daylong movement intervention on weight regain among older adults with obesity [32, 33]. Older adults aged 65–85 years with obesity (body mass index, BMI, 30–45 kg/m^2^) were recruited from a midsized city in the Southeastern United States. To be eligible, participants had to be low active (self‐reported ≤ 2 sessions of ≥ 30 min/week of moderate activity), non smoking, weight stable in the past 6 months (< 5% body weight change), and free of most chronic health conditions (e.g., diabetes, osteoporosis, cognitive impairment) [32]. A web‐based randomization tool was utilized to assign participants to each intervention group at a ratio of 1:1:1, stratified by sex and using random block sizes. All eligible participants were screened via telephone before completing a morning in‐person clinic visit after an overnight fast. A total of 183 participants were randomized (Figure 1) [33]. The Institutional Review Board of the Wake Forest School of Medicine approved all study‐related procedures. All participants provided written informed consent.

**FIGURE 1:**
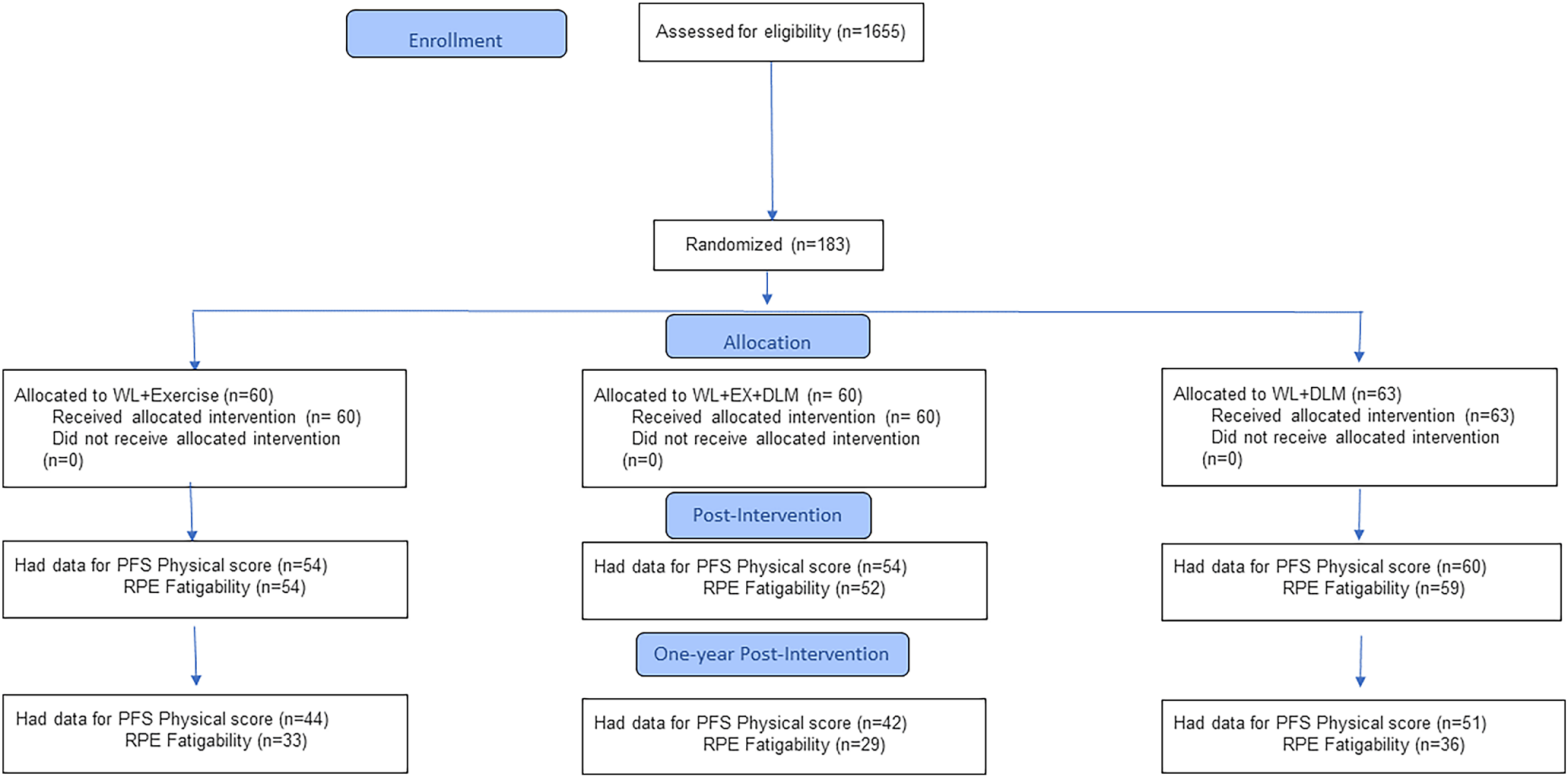
Modified CONSORT 2025 flowchart of participants with completed perceived physical fatigability measures at 6 and 18 months: EMPOWER. WL + EX = Weight loss plus aerobic exercise; WL + EX+DLM = Weight loss plus aerobic exercise plus daylong movement; WL + DLM = Weight loss plus daylong movement; PFS = Pittsburgh Fatigability Scale; RPE = Rating of Perceived Exertion. [Color figure can be viewed at wileyonlinelibrary.com]

### 2.1 Study Timeline

From October 2016 to January 2019, participants were recruited through newspaper and written advertisements and were enrolled in a total of six waves (*n* = 24–33 per wave) [33]. Participants completed assessments at baseline, 6 months (end of intensive intervention period), and 18 months (end of maintenance period). Final follow‐up completed in September 2020. Because of the COVID‐19 pandemic quarantine orders, waves 5 and 6 (*n* = 63) only completed self‐reported assessments (i.e., did not complete in‐person assessments) at 18 months.

### 2.2 Intervention

There were three intervention groups: (1) weight loss plus aerobic exercise (WL + EX), (2) weight loss plus daylong movement (WL + DLM), and (3) weight loss plus aerobic exercise plus daylong movement (WL + EX + DLM) (Table 1). All intervention groups received a Fitbit Alta to track their daily activity and had access to an mHealth smartphone application throughout the study period. The mHealth app provided group‐specific feedback derived from Fitbit data on progress toward physical activity goals. The app also facilitated ongoing asynchronous communication between group members.

**TABLE 1:** Protocol for each intervention component in EMPOWER.

Duration	Intervention period	Maintenance period	
	6 Months	3 Months	9 Months
Weight loss component	Caloric deficit (~400 kcal/day)	2× month group meetings with registered dietitian and behavioral change expert	Monthly email or phone call check‐in
	Weekly group meetings supervised by registered dietitian and behavioral change expert		
		Daily food intake logs	
	Daily food intake logs		
	Weekly weigh‐ins		
Aerobic exercise component	3 center‐based treadmill walk sessions	1 center‐based treadmill walk session	Monthly email or phone call check‐in
	Warm‐up [3–5 min]	Warm‐up [3–5 min]	
	Up to 40 min walking at 65%–70% heart rate reserve	Up to 40 min walking at 65%–70% heart rate reserve	
	Cool down [3–5 min]	Cool down [3–5 min]	
	1+ home‐based aerobic sessions	3+ home‐based aerobic sessions	
	13–15 Borg RPE intensity	13–15 Borg RPE intensity	
Daylong movement component	Focus on using mHealth and Fitbit to distribute activity across day and progressively increase step count	2× month group meetings	Monthly email or phone call check‐in
	Weekly group meetings		

*Note:* Participants in the Empowered with Movement to Prevent Weight Regain (EMPOWER) trial were randomized into one of three intervention groups: WL + EX, WL + DLM, or WL + EX+DLM. WL + EX consisted of weight loss and aerobic exercise components, WL + DLM consisted of weight loss plus daylong movement components, and WL + EX+DLM consisted of all three intervention components.

Regarding the weight loss goal, all three intervention groups were designed to elicit similar weight loss of 7%–10% during the first 6 months of the trial. Therefore, all intervention groups received an identical dietary modification component. Individual daily caloric intake goals were estimated to be ~400 kcal/day caloric deficit from weight maintenance. Participants attended weekly group meetings with a registered dietitian and behavioral change expert during the intensive intervention period; sessions focused on topics such as self‐regulation, mindful eating, and nutritional knowledge. Participants also completed daily food intake logs and had weekly body weight check‐ins during the group meetings. The final month of the intensive intervention period was focused on self‐management of weight loss.

Participants randomized to WL + EX and WL + EX + DLM engaged in the aerobic exercise (EX) intervention component. The goal was to progress toward achieving 200 min of moderate‐intensity physical activity across four to five structured exercise sessions per week. During the intensive intervention period, these groups were encouraged to participate in three treadmill walking sessions at the center plus at least one exercise session at home. During center‐based aerobic treadmill walking sessions, participants began with a 3– to 5‐min walking warm‐up, followed by a walking session at a target of 65%–70% of heart rate reserve. Duration of the walking sessions increased according to participants' ability with the goal of walking for 40 min by Week 6 of the program. Participants completed each session with a 3– to 5‐min walking cooldown and 5 min of flexibility stretching. Participants were monitored during each walking session by two heart rate readings; speed and grade during the walking session were adjusted to maintain work in the prescribed intensity zone. The EX participants were also provided with safety information on completing aerobic exercise on their own at an intensity of 13–15 on the Borg Rating of Perceived Exertion scale (Borg RPE, range 6–20).

Participants in WL + DLM and WL + EX + DLM engaged in the daylong movement component (DLM) and aimed to reduce long periods of inactivity by emphasizing frequent bouts of activity each day. Participants were encouraged to distribute their activities evenly across the day and to progressively improve their daily step count by 25% each week until they reached 10,000 steps per day. Weekly in‐person group sessions occurred during group dietary sessions and focused on providing guidance and motivation to achieve stepping goals. Participants were encouraged to use the provided Fitbit device and mHealth app to track progress.

During months 7–18, participants participated in a 3‐month transition period followed by a 9‐month maintenance period. During the maintenance period, all participants (regardless of intervention allocation) received monthly email or phone calls to encourage maintaining their activity and retention in the study (Table 1). For this analysis, we will collectively refer to the transition and maintenance period as the maintenance period.

### 2.3 Outcome

Perceived physical fatigability was measured at baseline, 6 months, and 18 months using two methods: (1) Pittsburgh Fatigability Scale (PFS) [34] and (2) Borg RPE after a 5‐min slow walking task (RPE Fatigability) [35]. These measures provide complementary assessments of fatigability as PFS is sensitive to change in lifestyle fatigability, whereas RPE Fatigability directly assesses fatigue associated with a low‐effort walking task [17]. PFS is a validated 10‐item questionnaire which asks participants to rate the level of physical and mental fatigue they expect or imagine they would feel immediately after completing each item [34, 36]. The PFS Mental score was not included in this analysis. Each item in PFS has a specific intensity and duration: (1) leisurely walk for 30 min, (2) brisk or fast walk for 1 h, (3) light household activity for 1 h, (4) heavy gardening or outdoor work for 1 h, (5) watching television for 2 h, (6) sitting quietly for 1 h, (7) moderate‐ to high‐intensity strength training for 30 min, (8) participating in a social activity for 1 h, (9) hosting a social event for 1 h, and (10) high‐intensity activity for 30 min. PFS scores range from 0 to 50; higher scores indicate greater fatigability. PFS has excellent convergent and concurrent validity in older adult populations [34]. Additionally, internal consistency and test–retest reliability are high [34]. PFS scores ≥ 15 indicate having more severe fatigability. A clinically meaningful difference in PFS score has been previously defined as 4 points [13]. For descriptive purposes, PFS scores were also classified into established severity strata: 0–4, 5–9, 10–14, 15–19, 20–24, and ≥ 25 [13, 37].

RPE Fatigability, previously validated in a healthy cohort of older adults [35], was measured immediately after a 5‐min treadmill walk at 2.0 mph and 0% grade. Immediately at the end of the 5‐min walk, participants were asked to rate their perceived effort using the Borg RPE scale. Scores range from 6 to 20 and higher scores indicate greater RPE Fatigability. For descriptive purposes, RPE Fatigability was classified as low (≤ 7), medium (8–9), or high (≥ 10) [35]. RPE Fatigability scores ≥ 10 indicates having more severe fatigability [35].

### 2.4 Covariates

Age, sex, race, and education level were self‐reported at baseline. Height and weight were measured at the clinical center using standardized equipment. BMI (kg/m^2^) was calculated using height and weight. Free‐living (continuous 7‐day wear on nondominant thigh) physical activity was measured at baseline, 6 months, and 18 months using the activPAL (PAL Technologies Ltd.), a three‐axis accelerometer and inclinometer validated for use among older adults [38, 39]. Mean daily step count and moderate‐vigorous physical activity (MVPA, min/day), defined as the time spent stepping ≥ 100 steps/min, were calculated [40]. Methods related to peak oxygen consumption and cost‐capacity ratio are described in online Supporting Information (Table S1).

### 2.5 Sample Size and Statistical Power

A priori sample size and power calculations estimated an 85% completion rate (51 completers per group), and 80% power provided the ability to detect effect sizes of 0.24–0.49 for the prespecified tertiary outcomes, which included fatigability [32]. Due to the COVID‐19 pandemic, attrition rates were higher than expected. Out of the 183 older adults with obesity randomized in EMPOWER, 168 participants had complete data for PFS and 165 had RPE Fatigability at baseline and 6 months. Of these, 137 and 98 participants had PFS and RPE Fatigability data at 18 months, respectively (Figure 1). Missingness differed by education level; therefore, all models were adjusted for education level.

### 2.6 Statistical Analysis

Main outcomes of EMPOWER, including weight and physical activity changes, as well as adherence, have been previously reported [33]. Participant baseline characteristics were described using mean ± standard deviation or frequencies (percent). We described changes in PFS and RPE Fatigability severity visually. We used linear mixed models (LMMs) to assess changes in fatigability over time. Separate LMMs were fitted for each outcome: continuous PFS and RPE Fatigability measured at baseline, 6 months, and 18 months. The models included fixed effects for intervention group, time (a categorical variable), and the time × intervention group interaction, while adjusting for age, sex, and education level. A compound symmetric covariance structure for repeated outcomes per person was selected based on AIC (Akaike information criterion) and BIC (Bayesian information criterion). We used the Satterthwaite approximation to compute denominator degrees of freedom for *F* or chi‐square tests. Model parameters were estimated using the restricted maximum likelihood (REML) method. In addition to testing the interaction effect between time and intervention group, we conducted pairwise comparisons between intervention groups for changes from baseline to 6 months and from 6 months to 18 months using linear contrasts; to account for multiple comparisons, Bonferroni correction was used. All hypotheses were tested as two‐tailed at a significance level of 0.05. To evaluate the potential impact of missing data due to the mandated quarantine orders during the COVID‐19 pandemic, we ran a sensitivity analysis excluding recruitment waves affected by clinic shutdowns (waves 5–6).

Additionally, we used a per‐protocol approach to assess change in fatigability outcomes among those who met weight loss goals. A total of 102 participants met the prespecified weight loss goal of at least 7% at 6 months (*n* = 66 did not meet weight loss goals, *n* = 15 did not complete intervention). We used the inverse probability weighting (IPW) method using a logistic regression model to address potential selection bias in our per‐protocol analysis. This approach allowed us to effectively balance the baseline covariates between those who did and did not meet weight loss goals. Weights identified from IPW were applied to LMMs for the sensitivity analysis. All analyses were conducted using SAS v9.4 (SAS Institute Inc.).

## 3 Results

On average, participants (*n* = 183) were 69.6 ± 4.3 years old with BMI of 35.6 ± 3.8 kg/m^2^. Overall, 77% were female and 74% were White non‐Hispanic (Table 2). At baseline, PFS score was 20.6 ± 7.6 and RPE Fatigability was 9.6 ± 1.9 (Table 2). There were no differences across intervention groups at baseline. Additionally, there was no significant difference in loss‐to‐follow‐up by intervention group or recruitment wave (*p* > 0.05).

**TABLE 2:** Baseline characteristics of participants in the Empowered with Movement to Prevent Weight Regain (EMPOWER) randomized clinical trial by intervention group.

	Total (*N* = 183)*	WL + EX (*n* = 60)	WL + DLM (*n* = 63)	WL + EX + DLM (*n* = 60)
Age, years	69.6 ± 4.2	70.2 ± 4.2	69.5 ± 4.6	69.1 ± 3.9
Female	141 (77.1)	48 (80.0)	47 (74.6)	46 (76.7)
White non‐Hispanic	136 (74.3)	45 (75.0)	49 (77.8)	42 (70.0)
Education				
High school	25 (13.7)	8 (13.3)	6 (9.5)	11 (18.3)
College	105 (57.4)	33 (55.0)	38 (60.3)	34 (56.7)
Postgraduate	53 (28.9)	19 (31.7)	19 (30.2)	15 (25.0)
Height, m	1.64 ± 0.09	1.62 ± 0.08	1.7 ± 0.09	1.65 ± 0.07
Weight, kg	96.3 ± 13.9	94.4 ± 14.1	97.3 ± 14.8	97.1 ± 13.0
BMI, kg/m^2^	35.6 ± 3.8	35.6 ± 3.8	35.5 ± 3.9	35.6 ± 3.7
Step count, /day	6412 ± 2401	6371 ± 2335	6380 ± 2523	6497 ± 2357
MVPA, min/day	9.5 ± 9.0	9.7 ± 9.7	10.0 ± 9.6	8.4 ± 7.7
PFS Physical score, 0–50	20.6 ± 7.6	21.5 ± 6.9	20.9 ± 7.4	19.4 ± 8.4
PFS Physical score ≥ 15	147 (80.3)	53 (88.3)	49 (77.8)	45 (75.0)
RPE Fatigability, 6–20	9.6 ± 1.9	9.4 ± 1.9	9.7 ± 1.9	9.7 ± 1.9
RPE Fatigability ≥ 10	87 (47.5)	24 (40.0)	31 (49.2)	32 (53.3)

*Note:* Values shown are mean ± standard deviation or *n* (%).

Abbreviations: MVPA, moderate‐vigorous physical activity; PFS, Pittsburgh Fatigability Scale; RPE, Borg Rating of Perceived Exertion; WL + DLM, weight loss plus daylong movement; WL + EX, weight loss plus aerobic exercise; WL + EX + DLM, weight loss plus aerobic exercise plus daylong movement.

* There were no significant differences at baseline by intervention groups (all *p* > 0.05).

Participants lost on average 8.4% ± 5.2% of their body weight between baseline and 6 months (range: 5% gain—21% loss); there were no differences between intervention groups. Additionally, MVPA more than doubled (9.5 min/day to 20.2 min/day), and participants took 1679 more steps/day at 6 months (Table S1). Relative peak oxygen consumption also improved overall by 1.6 mL kg^−1^ min^−1^ (Table S1). By 18 months, physical activity measures were more similar to baseline values, whereas body weight and peak oxygen consumption remained slightly improved from baseline (Table S1).

### 3.1 Pittsburgh Fatigability Scale Physical Score

Most participants (80%) reported being in the higher categories of PFS severity at baseline (≥ 15), with 26.2%, 20.8%, and 33.3% in the 15–19, 20–24, and ≥ 25 categories, respectively (Figure 2). At 6 months, 42% of participants (*n* = 35) moved out of the most severe PFS strata (≥ 25), and the number of participants in the lowest PFS severity strata (0–4) increased more than 300% (*n* = 3 at baseline to *n* = 13 post‐intervention). At 18 months, 24.1% of participants were classified into the most severe PFS severity strata. There were no differences across PFS severity strata between intervention groups at any time point (Figure 2).

**FIGURE 2:**
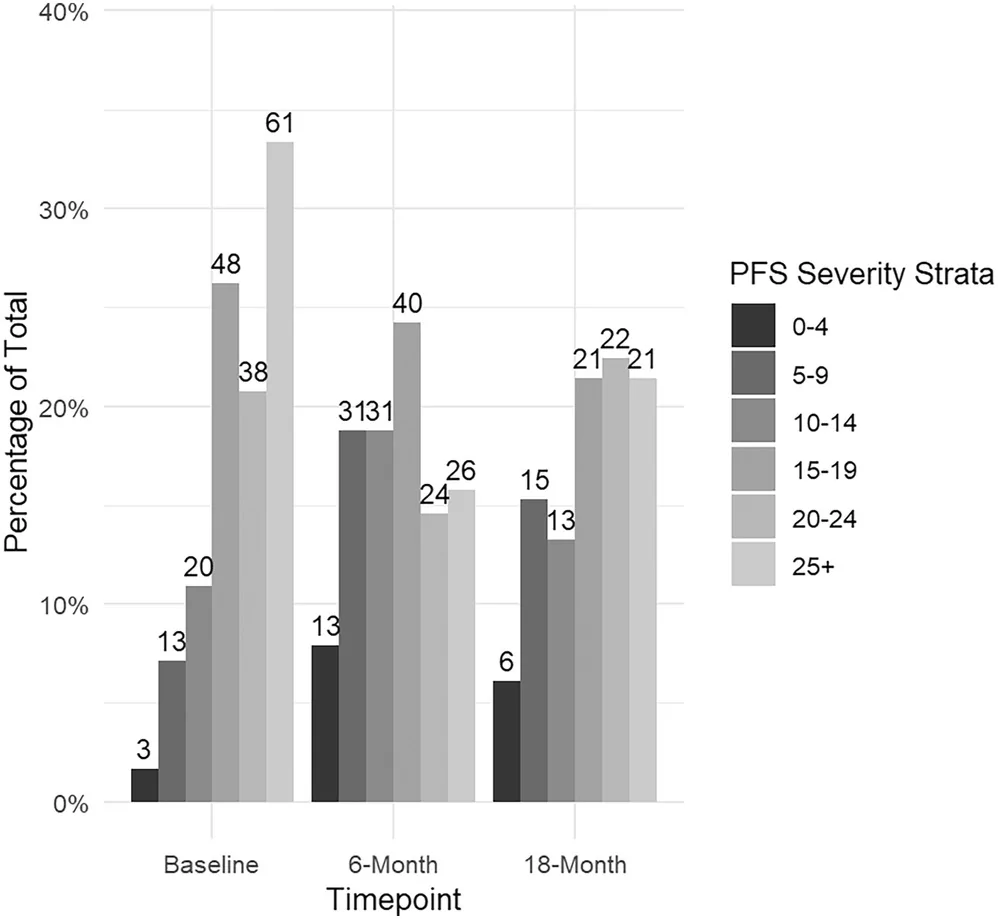
Classification of perceived physical fatigability severity at baseline, 6 months, and 18 months: EMPOWER. Figure shows the *n* of each Pittsburgh Fatigability Scale (PFS) Physical score severity strata at baseline, 6‐month, and 18‐month visits. PFS Physical scores range from 0 to 50, with a higher score indicating greater fatigability. Categories in PFS severity strata are as follows: 0–4 least severe, 5–9, 10–14, 15–19, 20–24, ≥ 25 most severe.

PFS score significantly improved over time, with an average 21% reduction (−4.4 points, *p* < 0.0001) from baseline to 6 months and 14% reduction (−3.0 points, *p* < 0.001) from baseline to 18 months after adjustment for age, sex, education level, intervention group, and time × intervention group (Table 3). There were significant improvements within WL + EX, WL + DLM, and WL + EX + DLM groups from baseline to 6 months after adjustment (Figure 3; −4.4, −6.1, and −4.9, respectively). However, the overall time × intervention group interaction effect was not significant, indicating no between‐group differences in improvement of PFS score over time (*p* = 0.83). Restricting the analysis to recruitment waves 1–4 (who had in‐person assessments prior to COVID‐related closures) still showed no significant interaction effect.

**TABLE 3:** Estimates of fixed effects from linear mixed models estimating change in fatigability during EMPOWER study (*N* = 183).

	PFS Physical score estimate (SE)	RPE Fatigability estimate (SE)
Intercept	10.4 (6.5)	4.0 (1.8)*
6 Months	−4.4 (1.5)*	−0.02 (0.4)
18 Months	−3.1 (1.4)*	−0.5 (0.4)
Intervention group		
WL + DLM	REF	REF
WL + EX	0.39 (1.4)	−0.40 (0.4)
WL + EX + DLM	−1.46 (1.4)	−0.02 (0.4)
6 Months × WL + EX	−1.68 (2.1)	−0.17 (0.5)
18 Months × WL + EX	0.61 (2.2)	0.79 (0.6)
6 Months × WL + EX + DLM	−0.38 (2.1)	−0.90 (0.5)
18 Months × WL + EX + DLM	1.22 (2.2)	−0.09 (0.6)

*Note:* Models were adjusted for age, sex, and education level. The time × intervention group interaction effect was not significant in either linear mixed model (PFS model: *F*‐statistic = 0.36, *p* = 0.83; RPE model: *F*‐statistic = 1.35, *p* = 0.24).

Abbreviations: PFS, Pittsburgh Fatigability Scale; RPE, Borg Rating of Perceived Exertion; SE, standard error; WL + DLM, weight loss plus daylong movement; WL + EX, weight loss plus aerobic exercise; WL + EX + DLM, weight loss plus aerobic exercise plus daylong movement.

* *p* < 0.05.

**FIGURE 3:**
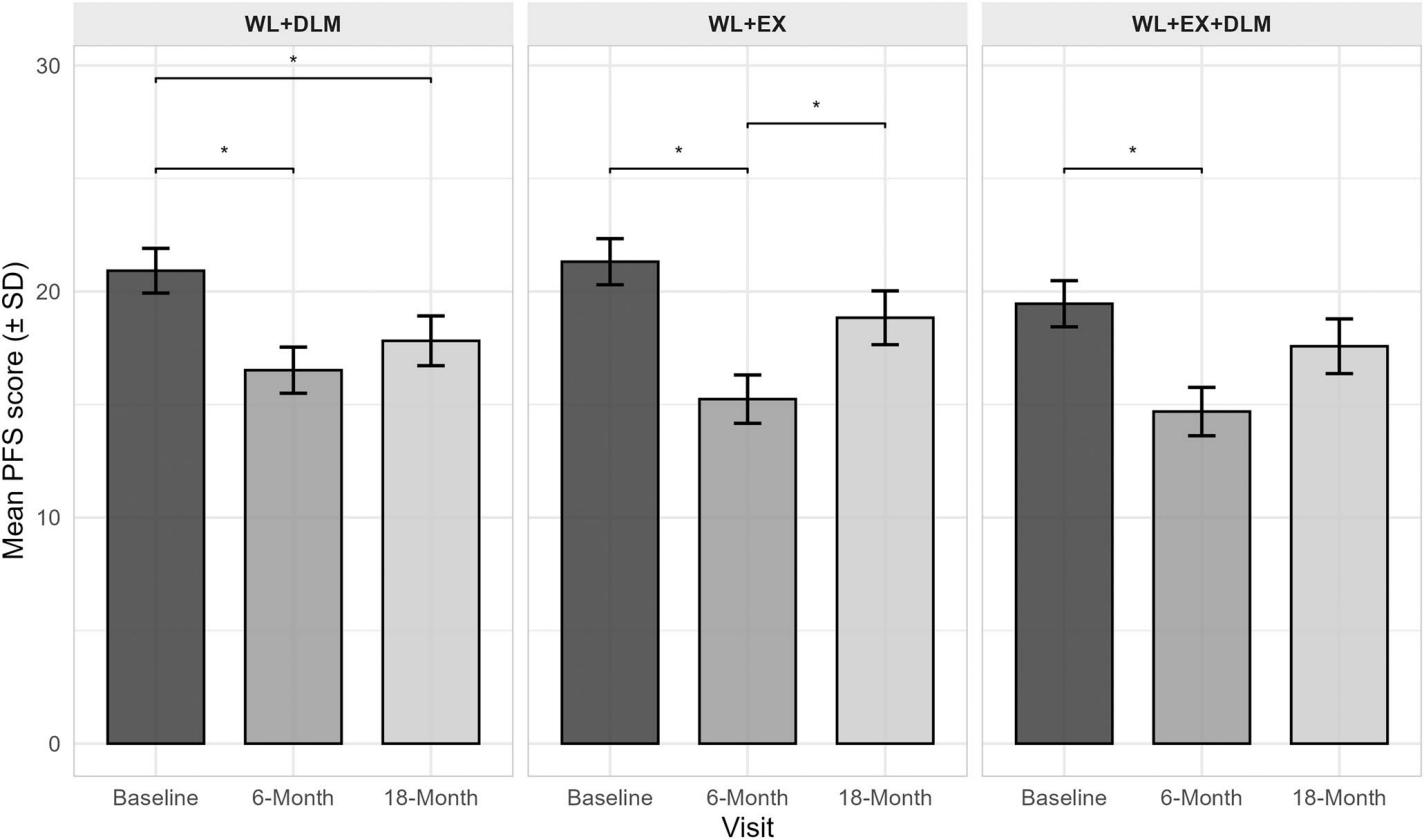
Adjusted mean (± standard deviation) of Pittsburgh Fatigability Scale (PFS) physical scores by intervention group over time. *p < 0.05. WL + DLM = Weight loss plus daylong movement; WL + EX = Weight loss plus aerobic exercise; WL + EX + DLM = Weight loss plus aerobic exercise plus daylong movement. PFS scores were measured at baseline, 6‐month, and 18‐month visits. Means are adjusted for age, sex, and education level.

### 3.2 RPE Fatigability

At baseline, 47.5% of participants had high RPE Fatigability (*n* = 87; ≥ 10 RPE), while only 10.9% had low RPE Fatigability (*n* = 20; ≤ 7 RPE) (Figure 4). At 6 months, 37.6% of participants reported medium and high RPE Fatigability (*n* = 62 each), respectively. At 18 months, 49% of participants who completed the 5‐min slow walking task had high RPE Fatigability (*n* = 48). There were no differences in RPE Fatigability severity by intervention group at any time point (Figure 4). When LMMs were repeated using RPE Fatigability as the outcome, there was no significant effect by time (*p* = 0.23), intervention group (*p* = 0.36), or time × intervention group (*p* = 0.24) (Table 3).

**FIGURE 4:**
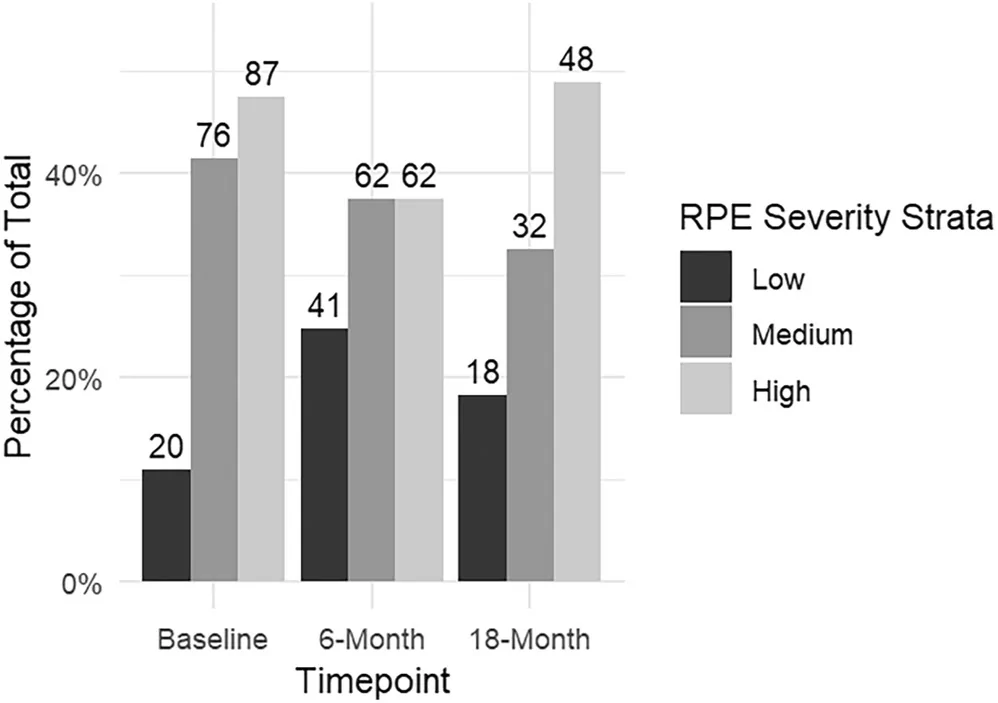
Classification of RPE Fatigability severity at baseline, 6 months, and 18 months: EMPOWER. Values in figure represent *n* of each RPE Fatigability (measuring via the Borg Rating of Perceived Exertion after the end of a 5‐min treadmill walk at 2.0 mph) range from 6 to 20, with a higher score indicating greater fatigability. Categories of RPE Fatigability severity are as follows: low (6–7), medium (8–9), high (≥ 10).

### 3.3 Sensitivity Analysis: Per‐Protocol Approach

A total of 102 participants met the ≥ 7% weight loss goal. At baseline, those who met the goal were more likely to be White non‐Hispanic and have more severe PFS score (≥ 15) (Table S2). PFS score significantly improved over time (*p* < 0.01). Compared to the baseline score, PFS score was on average 2.2 points lower at 6 months (*p* = 0.03) and 0.2 points lower at 18 months (*p* = 0.82), after adjustment for age, sex, education level, intervention group, and time × intervention group. There was no effect of intervention group on PFS score (*p* = 0.96), and the time × intervention group interaction effect was not significant (*p* = 0.09). There were no significant associations when RPE Fatigability was the outcome (Table 4).

**TABLE 4:** Estimates of fixed effects from linear mixed models estimating change in fatigability among those who met weight loss goals, EMPOWER Study (*N* = 102).

	PFS Physical score estimate (SE)	RPE Fatigability estimate (SE)
Intercept	−0.28 (14.9)	1.7 (3.6)
6 Months	−2.2 (1.0)*	−0.2 (0.3)
18 Months	−0.2 (1.1)	−0.03 (0.3)
Intervention group		
WL + DLM	REF	REF
WL + EX	2.2 (2.0)	0.07 (0.5)
WL + EX + DLM	2.7 (2.0)	0.29 (0.5)
6 Months × WL + EX	−3.8 (1.8)	−0.4 (0.5)
18 Months × WL + EX	−2.0 (2.0)	0.24 (0.6)
6 Months × WL + EX + DLM	−2.8 (1.8)	−0.7 (0.5)
18 Months × WL + EX + DLM	−4.0 (1.8)	−1.0 (0.6)

*Note:* Models were adjusted for age, sex, and education level. The time × intervention group interaction effect was not significant in either linear mixed model (PFS model: *F*‐statistic = 2.02, *p* = 0.09; RPE model: *F*‐statistic = 1.35, *p* = 0.25).

Abbreviations: PFS, Pittsburgh Fatigability Scale; RPE, Borg Rating of Perceived Exertion; SE, standard error; WL + DLM, weight loss plus daylong movement; WL + EX, weight loss plus aerobic exercise; WL + EX+DLM, weight loss plus aerobic exercise plus daylong movement.

* *p* < 0.05.

## 4 Discussion

In a randomized clinical trial of older adults with obesity and high perceived physical fatigability, we observed clinically relevant improvements in PFS scores, but not RPE Fatigability, over a 6‐month intensive weight loss intervention. The improvements in PFS scores were similar across the three intervention groups over the duration of the intervention, and these improvements persisted after the maintenance period, suggesting that multiple physical activity intervention strategies can be considered in future translational interventions to lower fatigability among older adults with obesity. Our work demonstrates that fatigability, which is a characteristic of aging implicated in the disability pathway, is modifiable and should be targeted to increase the healthspan of community‐dwelling older adults with obesity.

Prior work has shown that physical activity interventions can lower fatigability [26, 27, 28, 29]. Qiao et al. examined the effects of a 6‐ to 14‐week exercise intervention among breast cancer survivors (mean age: 54 years) and found a 4‐point clinically meaningful improvement in PFS scores from baseline to post‐intervention within the intervention group [26]. Additionally, three other studies saw smaller but significant improvements in fatigability from pre‐ to post‐intervention, but only Qiao et al. found a statistically significant difference between exercise intervention versus no exercise control group [27, 28, 29]. We observed a clinically meaningful improvement in PFS scores at a magnitude similar to Qiao et al. during the intervention period. Our findings provide novel insights into the effectiveness of weight loss and physical activity interventions for lowering fatigability. In particular, the aerobic exercise protocol for the WL + EX and WL + EX + DLM intervention groups in our present study was clearly described in terms of frequency, intensity, duration, and type as these groups progressed to achieve 200 min/week of moderate‐intensity aerobic activity. While other studies also aimed to improve activity via increasing MVPA, no studies provided a replicable protocol as they failed to define all of the following components: frequency, intensity, duration, and activity type [26, 27, 28, 29]. Though more research is indicated to determine the optimal dose–response association between physical activity and fatigability, we demonstrated that encouraging older adults with obesity to reach at least 150 min/week of moderate‐intensity aerobic exercise (i.e., meet the 2018 US physical activity aerobic guidelines) can lead to meaningful changes in fatigability measured using PFS. Additionally, investigating the effects of the WL + DLM and WL + EX + DLM provided novelty as the DLM component aimed to naturally increase activity levels through an emphasis on frequent bouts of physical activity. This intervention was just as effective in lowering fatigability among older adults with obesity. Taken together, we demonstrated that there may be multiple strategies that effectively lower fatigability among older adults with obesity. Future work can leverage this finding by administering tailored interventions that intervene on different dimensions of physical activity behaviors to fit the needs of the older adult.

Higher body weight is associated with greater fatigability [11, 12]. Nicklas et al. found that, while both exercise alone and exercise plus caloric restriction led to significant increases in vitality (i.e., decreases in fatigue levels) among older adults with obesity, exercise plus caloric restriction had an almost twofold greater benefit to vitality than exercise alone [31]. Because there was no weight loss only or activity only intervention group in EMPOWER, we cannot rule out the improvements in PFS scores are due to weight loss. However, we attempted to address this limitation by performing a sensitivity analysis only among those with ≥ 7% weight loss during the first 6 months of the trial. Interestingly, while PFS scores did significantly improve among those who met ≥ 7% weight loss, they had roughly 50% less improvement (2.2 vs. 4.4 points) than results that included all EMPOWER study participants. This finding contradicts the hypothesis that greater weight loss is associated with greater benefits in fatigability. Our findings are strengthened by use of IPW to account for selection bias and suggest the association between body composition and fatigability may be nuanced and in need of further investigation. Further, given that weight changes and fatigability are both associated with energy expenditure, future work is indicated to determine whether change in energy expenditure drives changes in fatigability. For example, a net zero change in energy expenditure despite significant weight loss may explain the smaller magnitude of improvement observed in the sensitivity analysis.

To our knowledge, we are the first study to examine the long‐term impact of different physical activity interventions in the context of weight loss on fatigability. While PFS scores did slightly increase during the 12‐month maintenance period, they were still on average 3 points lower than baseline PFS values after covariate adjustment. Prospective data describing age‐related changes in fatigability are lacking. Therefore, we are unable to compare the changes observed during the maintenance period to expected changes due to normal aging. Nevertheless, our results suggest that the benefits achieved through the intervention were largely maintained. Additionally, as the intensive intervention had ended, less adherence to physical activity and diet behaviors may have contributed to the slight increases in PFS scores from 6 months to 18 months. More work is indicated to understand factors associated with maintaining fatigability levels among older adults with obesity.

We found that PFS scores but not RPE Fatigability improved during the EMPOWER intervention and maintenance periods. Lack of significant improvements may be in part due to an anchor effect, where participants may be more likely to rate their fatigue using a value that corresponds to an anchor phrase. Mean RPE Fatigability was around 9 points at each time point, which is anchored to the phrase “very light.” Thus, the task seemed to have been too easy to elicit adequate fatigue, and this may translate to less meaningful changes in older adults' perceptions. In a cohort of > 800 healthy older adults, the correlation between RPE Fatigability and PFS was 0.33, suggesting there is weak to moderate overlap between measures [17]. The PFS, which measures fatigability across activities of varying intensities (i.e., light to high) and durations, is likely to be more sensitive to change during a physical activity intervention because it assesses tasks performed at higher intensities. Assessing and intervening on PFS, therefore, may better translate to a meaningful change in quality of life. Further, implementing the use of PFS in clinical settings could identify at‐risk older adults who would benefit from physical activity interventions.

Our work has a few limitations. First, due to the study design, we were unable to understand the effects of different physical activity interventions independent of weight loss, though our per‐protocol sensitivity analysis showed smaller improvements in PFS scores among those with greater weight loss. Given the well‐described intervention protocol, our work still informs future translational physical activity interventions. Additionally, our sample was relatively healthy and mostly non‐Hispanic White, limiting our generalizability to a broader older adult population. Study attrition rates were slightly higher than a priori estimates; this likely impacted our power to detect between‐group differences in perceived physical fatigability. While the end of the intervention coincided with COVID‐19 stay‐at‐home mandates, sensitivity analyses showed that this did not impact our study findings.

A key strength of this study is the use of two well‐validated measures of perceived physical fatigability [34, 35]. Additionally, because of the 1‐year follow‐up period, we were able to assess the long‐term effect of the intervention on fatigability. Finally, older adults with obesity represent a population at risk for both greater fatigability and functional decline. Our work highlights that targeting this population in future translational interventions to lower fatigability may have a meaningful public health impact.

## 5 Conclusion

We revealed that weight loss plus physical activity interventions elicited clinically meaningful reduction of perceived physical fatigability measured by the PFS among older adults with obesity and these benefits were largely maintained after 18 months. Both encouraging older adults to achieve 150 min/week of moderate‐vigorous aerobic exercise and improving physical activity distribution throughout the day are effective strategies to lower fatigability. As large gaps between lifespan and healthspan still remain, lowering fatigability through physical activity interventions may reduce this gap and improve the quality of life for older adults with obesity.

## Author Contributions

R.E.G. conceptualized the research question, completed the data analysis, drafted the initial manuscript, and reviewed and revised the manuscript. N.W.G. supervised the manuscript development and reviewed and revised the manuscript. J.F., C.K., B.R.‐W., C.E.K., and B.J.N. helped interpret results and reviewed and revised the manuscript. J.F. and B.J.N. conceptualized and conducted the EMPOWER study.

## Funding

The parent study was funded by the National Institutes of Health and National Institute on Aging (R01 AG051624). Dr. Jason Fanning is also supported by the Wake Forest University Claude D. Pepper Older Americans Independence Center (P30‐AG21332). Additionally, the NIA Pittsburgh Claude D. Pepper Older Americans Independence Center, Research Registry and Developmental Pilot Grant (P30 AG024827), and the Intramural Research Program, NIA supported Dr. Nancy W. Glynn to develop the Pittsburgh Fatigability Scale. Dr. Reagan E. Garcia is supported by the T32 Training in Epidemiology of Aging Program (T32 AG000181 PI: Dr. Strotmeyer).

## Conflicts of Interest

The authors declare no conflicts of interest.

## Supporting information


**Table S1:** Intervention‐related metrics at baseline 6 months, and 18 months: EMPOWER.
**Table S2:** Comparison of baseline characteristics between those who met weight loss goal of at least 7% bodyweight reduction vs. those who did not meet weight loss goal, *N* = 168.

## Data Availability

The data that support the findings of this study are available on request from the corresponding author. The data are not publicly available due to privacy or ethical restrictions.

## References

[1] A. Garmany and A. Terzic , “Global Healthspan‐Lifespan Gaps Among 183 World Health Organization Member States,” JAMA Network Open 7, no. 12 (2024): e2450241. 10.1001/jamanetworkopen.2024.50241 PMC11635540 39661386

[2] N. B. Alexander , G. E. Taffet , F. M. Horne , et al., “Bedside‐to‐Bench Conference: Research Agenda for Idiopathic Fatigue and Aging,” Journal of the American Geriatrics Society 58, no. 5 (2010): 967–975. 10.1111/j.1532-5415.2010.02811.x PMC4540791 20722821

[3] R. J. Valentine , E. McAuley , V. J. Vieira , et al., “Sex Differences in the Relationship Between Obesity, C‐Reactive Protein, Physical Activity, Depression, Sleep Quality and Fatigue in Older Adults,” Brain, Behavior, and Immunity 23, no. 5 (2009): 643–648. 10.1016/j.bbi.2008.12.003 19133324

[4] E. Lærum‐Onsager , T. Brovold , A. Bergland , A. H. Pripp , and A. Bye , “Associations Between Health‐Related Quality of Life, Body Mass Index, Health Status and Sociodemographic Variables in Geriatric Patients and Non‐Hospitalized Older People: A Comparative Cross‐Sectional Study,” Nutrition and Health 26, no. 2 (2020): 141–150. 10.1177/0260106020909047 32297563

[5] L. DiPietro , Y. Jin , S. Talegawkar , and C. E. Matthews , “The Joint Associations of Weight Status and Physical Activity With Mobility Disability: The NIH‐AARP Diet and Health Study,” International Journal of Obesity (Lond) 43, no. 9 (2019): 1830–1838. 10.1038/s41366-018-0294-8 PMC8491301 30575803

[6] Y. Su , B. B. Cochrane , S.‐Y. Yu , K. Reding , J. R. Herting , and O. Zaslavsky , “Fatigue in Community‐Dwelling Older Adults: A Review of Definitions, Measures, and Related Factors,” Geriatric Nursing 43 (2022): 266–279. 10.1016/j.gerinurse.2021.12.010 34963072

[7] B. A. Eldadah , “Fatigue and Fatigability in Older Adults,” PM & R: The Journal of Injury, Function, and Rehabilitation 2, no. 5 (2010): 406–413. 10.1016/j.pmrj.2010.03.022 20656622

[8] K. R. LaSorda , T. Gmelin , A. L. Kuipers , et al., “Epidemiology of Perceived Physical Fatigability in Older Adults: The Long Life Family Study,” Journals of Gerontology. Series A, Biological Sciences and Medical Sciences 75, no. 9 (2020): e81–e88. 10.1093/gerona/glz288 PMC7494027 31828303

[9] M. T. Prochaska , H. Zhang , C. Alavi , and D. O. Meltzer , “Fatigability: A New Perspective on and Patient‐Centered Outcome Measure for Patients With Anemia,” American Journal of Hematology 95, no. 7 (2020): E166–E169. 10.1002/ajh.25803 PMC7605445 32291785

[10] M. T. Prochaska , H. Zhang , R. Babajide , and D. O. Meltzer , “The Effect of Red Blood Cell Transfusion on Fatigability After Hospital Discharge,” Blood Advances 4, no. 22 (2020): 5690–5697. 10.1182/bloodadvances.2020003364 PMC7686894 33211825

[11] R. Cooper , M. Popham , A. J. Santanasto , R. Hardy , N. W. Glynn , and D. Kuh , “Are BMI and Inflammatory Markers Independently Associated With Physical Fatigability in Old Age?,” International Journal of Obesity (Lond) 43, no. 4 (2019): 832–841. 10.1038/s41366-018-0087-0 PMC6477893 29795469

[12] P. Martinez‐Amezcua , E. M. Simonsick , A. A. Wanigatunga , et al., “Association Between Adiposity and Perceived Physical Fatigability in Mid‐ to Late Life,” Obesity (Silver Spring) 27, no. 7 (2019): 1177–1183. 10.1002/oby.22500 PMC6591054 31127707

[13] E. M. Simonsick , J. A. Schrack , A. J. Santanasto , S. A. Studenski , L. Ferrucci , and N. W. Glynn , “Pittsburgh Fatigability Scale: One‐Page Predictor of Mobility Decline in Mobility‐Intact Older Adults,” Journal of the American Geriatrics Society 66, no. 11 (2018): 2092–2096. 10.1111/jgs.15531 PMC6322394 30315707

[14] N. W. Glynn , T. Gmelin , S. W. Renner , et al., “Perceived Physical Fatigability Predicts All‐Cause Mortality in Older Adults,” Journals of Gerontology. Series A, Biological Sciences and Medical Sciences 77, no. 4 (2022): 837–841. 10.1093/gerona/glab374 PMC8974332 34908118

[15] F. Liu , A. A. Wanigatunga , M. Zampino , et al., “Association of Mitochondrial Function, Substrate Utilization, and Anaerobic Metabolism With Age‐Related Perceived Fatigability,” Journals of Gerontology. Series A, Biological Sciences and Medical Sciences 76, no. 3 (2021): 426–433. 10.1093/gerona/glaa201 PMC8355455 32803242

[16] J. A. Schrack , A. A. Wanigatunga , V. Zipunnikov , P.‐L. Kuo , E. M. Simonsick , and L. Ferrucci , “Longitudinal Association Between Energy Regulation and Fatigability in Mid‐to‐Late Life,” Journals of Gerontology. Series A, Biological Sciences and Medical Sciences 75, no. 9 (2020): e74–e80. 10.1093/gerona/glaa011 PMC7494036 31942600

[17] E. L. Gay , P. M. Coen , S. Harrison , et al., “Sex Differences in the Association Between Skeletal Muscle Energetics and Perceived Physical Fatigability: The Study of Muscle, Mobility and Aging (SOMMA),” Geroscience 47, no. 2 (2025): 1999–2013. 10.1007/s11357-024-01373-z PMC11979008 39436549

[18] R. E. Garcia , T. L. Blackwell , D. E. Forman , et al., “Role of Walking Energetics and Perceived Fatigability Differs by Gait Speed: The Study of Muscle, Mobility and Aging (SOMMA),” Journals of Gerontology. Series A, Biological Sciences and Medical Sciences 79, no. 9 (2024): glae187. 10.1093/gerona/glae187 PMC11341985 39066507

[19] J. H. P. M. VAN DER Velde , A. Koster , J. D. VAN DER Berg , et al., “Sedentary Behavior, Physical Activity, and Fitness‐The Maastricht Study,” Medicine and Science in Sports and Exercise 49, no. 8 (2017): 1583–1591. 10.1249/MSS.0000000000001262 28319587

[20] J. A. Schrack , P.‐L. Kuo , A. A. Wanigatunga , et al., “Active‐to‐Sedentary Behavior Transitions, Fatigability, and Physical Functioning in Older Adults,” Journals of Gerontology. Series A, Biological Sciences and Medical Sciences 74, no. 4 (2019): 560–567. 10.1093/gerona/gly243 PMC6417447 30357322

[21] Y. S. Qiao , T. Gmelin , S. W. Renner , et al., “Evaluation of the Bidirectional Relations of Perceived Physical Fatigability and Physical Activity on Slower Gait Speed,” Journals of Gerontology. Series A, Biological Sciences and Medical Sciences 76, no. 10 (2021): e237–e244. 10.1093/gerona/glaa281 PMC8436994 33170216

[22] K. D. Moored , Y. S. Qiao , R. M. Boudreau , et al., “Prospective Associations Between Physical Activity and Perceived Fatigability in Older Men: Differences by Activity Type and Baseline Marital Status,” Journals of Gerontology. Series A, Biological Sciences and Medical Sciences 77, no. 12 (2022): 2498–2506. 10.1093/gerona/glac030 PMC9799181 35134905

[23] Y. S. Qiao , K. D. Moored , R. M. Boudreau , et al., “Changes in Objectively Measured Physical Activity Are Associated With Perceived Physical and Mental Fatigability in Older Men,” Journals of Gerontology. Series A, Biological Sciences and Medical Sciences 77, no. 12 (2022): 2507–2516. 10.1093/gerona/glac082 PMC9799193 35385877

[24] A. A. Wanigatunga , E. M. Simonsick , V. Zipunnikov , et al., “Perceived Fatigability and Objective Physical Activity in Mid‐ to Late‐Life,” Journals of Gerontology. Series A, Biological Sciences and Medical Sciences 73, no. 5 (2018): 630–635. 10.1093/gerona/glx181 PMC5905638 29028920

[25] J. L. Graves , Y. S. Qiao , K. D. Moored , et al., “Profiles of Accelerometry‐Derived Physical Activity Are Related to Perceived Physical Fatigability in Older Adults,” Sensors (Basel) 21, no. 5 (2021): 1718. 10.3390/s21051718 PMC7958607 33801352

[26] Y. Qiao , G. J. van Londen , J. W. Brufsky , et al., “Perceived Physical Fatigability Improves After an Exercise Intervention Among Breast Cancer Survivors: A Randomized Clinical Trial,” Breast Cancer 29, no. 1 (2022): 30–37. 10.1007/s12282-021-01278-1 34328623

[27] M. K. Jang , J. Han , S. H. Kim , Y. H. Ko , S. Y. Kim , and S. Kim , “Comparison of Fatigue and Fatigability Correlates in Korean Breast Cancer Survivors and Differences in Associations With Anxiety, Depression, Sleep Disturbance, and Endocrine Symptoms: A Randomized Controlled Trial,” BMC Cancer 21, no. 1 (2021): 855. 10.1186/s12885-021-08575-0 PMC8311955 34311713

[28] K. J. Collins , J. A. Schrack , J. M. VanSwearingen , et al., “Randomized Controlled Trial of Exercise to Improve Walking Energetics in Older Adults,” Innovation in Aging 2, no. 3 (2018): igy022. 10.1093/geroni/igy022 PMC6177056 30480143

[29] A. J. Hoffman , R. A. Brintnall , B. A. Given , A. von Eye , L. W. Jones , and J. K. Brown , “Using Perceived Self‐Efficacy to Improve Fatigue and Fatigability in Postsurgical Lung Cancer Patients: A Pilot Randomized Controlled Trial,” Cancer Nursing 40, no. 1 (2017): 1–12. 10.1097/NCC.0000000000000378 PMC5086324 27135752

[30] B. J. Nicklas , D. P. Beavers , S. L. Mihalko , G. D. Miller , R. F. Loeser , and S. P. Messier , “Relationship of Objectively‐Measured Habitual Physical Activity to Chronic Inflammation and Fatigue in Middle‐Aged and Older Adults,” Journals of Gerontology. Series A, Biological Sciences and Medical Sciences 71, no. 11 (2016): 1437–1443. 10.1093/gerona/glw131 PMC5055652 27382039

[31] B. J. Nicklas , T. E. Brinkley , D. K. Houston , et al., “Effects of Caloric Restriction on Cardiorespiratory Fitness, Fatigue, and Disability Responses to Aerobic Exercise in Older Adults With Obesity: A Randomized Controlled Trial,” Journals of Gerontology. Series A, Biological Sciences and Medical Sciences 74, no. 7 (2019): 1084–1090. 10.1093/gerona/gly159 PMC6580693 29982294

[32] J. Fanning , M. T. Opina , I. Leng , M. F. Lyles , B. J. Nicklas , and W. J. Rejeski , “Empowered With Movement to Prevent Obesity & Weight Regain (EMPOWER): Design and Methods,” Contemporary Clinical Trials 72 (2018): 35–42. 10.1016/j.cct.2018.07.010 30026128

[33] J. Fanning , W. J. Rejeski , I. Leng , et al., “Intervening on Exercise and Daylong Movement for Weight Loss Maintenance in Older Adults: A Randomized, Clinical Trial,” Obesity (Silver Spring) 30, no. 1 (2022): 85–95. 10.1002/oby.23318 PMC8711609 34932885

[34] N. W. Glynn , A. J. Santanasto , E. M. Simonsick , et al., “The Pittsburgh Fatigability Scale for Older Adults: Development and Validation,” Journal of the American Geriatrics Society 63, no. 1 (2015): 130–135. 10.1111/jgs.13191 PMC4971882 25556993

[35] E. M. Simonsick , J. A. Schrack , N. W. Glynn , and L. Ferrucci , “Assessing Fatigability in Mobility‐Intact Older Adults,” Journal of the American Geriatrics Society 62, no. 2 (2014): 347–351. 10.1111/jgs.12638 PMC3947865 24417536

[36] S. W. Renner , T. M. Bear , P. J. Brown , et al., “Validation of Perceived Mental Fatigability Using the Pittsburgh Fatigability Scale,” Journal of the American Geriatrics Society 69, no. 5 (2021): 1343–1348. 10.1111/jgs.17017 PMC8127403 33469914

[37] J. A. Schrack , E. M. Simonsick , and N. W. Glynn , “Fatigability: A Prognostic Indicator of Phenotypic Aging,” Journals of Gerontology. Series A, Biological Sciences and Medical Sciences 75, no. 9 (2020): e63–e66. 10.1093/gerona/glaa185 PMC7749190 32936913

[38] M. W. O'Brien , Y. Wu , J. L. Petterson , N. W. Bray , and D. S. Kimmerly , “Validity of the ActivPAL Monitor to Distinguish Postures: A Systematic Review,” Gait & Posture 94 (2022): 107–113. 10.1016/j.gaitpost.2022.03.002 35276456

[39] Y. Wu , J. L. Petterson , N. W. Bray , D. S. Kimmerly , and M. W. O'Brien , “Validity of the activPAL Monitor to Measure Stepping Activity and Activity Intensity: A Systematic Review,” Gait & Posture 97 (2022): 165–173. 10.1016/j.gaitpost.2022.08.002 35964334

[40] C. Tudor‐Locke , H. Han , E. J. Aguiar , et al., “How Fast Is Fast Enough? Walking Cadence (Steps/Min) as a Practical Estimate of Intensity in Adults: A Narrative Review,” British Journal of Sports Medicine 52, no. 12 (2018): 776–788. 10.1136/bjsports-2017-097628 PMC6029645 29858465

