# Supplementary material for: Weight Loss and Physical Activity Reduces Perceived Physical Fatigability Among Older Adults With Obesity
Source: Obesity (Silver Spring). 2026 Sep 9;34(11):2061–71. doi: 10.1002/oby.70298 (PMC13716182; doi:10.1002/oby.70298)
Supplement: Supplementary file 1 [file OBY-34-2061-s001.pdf]

Supplemental Material For: Weight Loss and Physical Activity Reduces Perceived Physical Fatigability Among Older Adults with Obesity

Authors: Reagan E. Garcia, PhD, MPH (1), Jason Fanning, PhD (2), Chaeryon Kang, PhD (3,4), Bonny Rockette-Wagner, PhD (1), Christopher E. Kline, PhD (5), Barbara J. Nicklas, PhD (6), Nancy W. Glynn, PhD (1)

Affiliations:

1. Department of Epidemiology, University of Pittsburgh, School of Public Health. Pittsburgh, PA.
2. Department of Health and Exercise Sciences, Wake Forest University, Winston-Salem, NC.
3. Department of Psychiatry, University of Pittsburgh, School of Medicine. Pittsburgh, PA.
4. Department of Biostatistics and Health Data Science, School of Public Health, University of Pittsburgh, Pittsburgh, PA.
5. Department of Health and Human Development, University of Pittsburgh School of Education. Pittsburgh, PA.
6. Department of Internal Medicine, Section on Gerontology and Geriatric Medicine, Wake Forest University School of Medicine. Winston-Salem, NC.

Keywords: fatigue, clinical trials, aging

Contact Information:

Nancy W. Glynn, PhD
epidnwg@pitt.edu
University of Pittsburgh School of Public Health
Department of Epidemiology, Center for Aging and Population Health
130 DeSoto Street, 5120 Public Health, Pittsburgh PA 15261

Clinical Trial Registration: ClinicalTrials.gov identifier: NCT02923674

Table S1: Intervention-related Metrics at Baseline, 6 Months, and at 18 Months: EMPOWER

	Baseline N=183	6 Month N=168	18 Month N=139	p-trend
**Weight, kg**	96.3 ± 13.9	88.3 ± 13.9	88.0 ± 13.8	<0.0001
**Body mass index, kg/m^2**	35.6 ± 3.8	32.6 ± 3.9	32.9 ± 4.1	<0.0001
**Step count, /day**	6412 ± 2401	8091 ± 3267	6475 ± 2566	<0.0001
**MVPA, min/day**	9.5 ± 9.0	20.2 ± 19.9	9.1 ± 12.2	<0.0001
**VO_2peak, $mL \cdot min^{-1}$**				0.13
**VO_2peak, $mL \cdot kg^{-1} \cdot min^{-1}$**	19.6 ± 4.1	21.2 ± 4.7	20.1 ± 4.3	0.002
**Cost-capacity ratio**	58.3 ± 15.6	50.8 ± 16.4	54.1 ± 14.6	<0.0001
**PFS Physical score, 0-50**	20.6 ± 7.6	15.5 ± 8.1	18.1 ± 8.3	<0.0001
**PFS Physical score ≥15**	147 (80.3)	91 (54.2)	91 (66.4)	<0.0001
**RPE Fatigability, 6-20**	9.6 ± 1.9	9.2 ± 1.9	9.4 ± 2.7	0.27
**RPE Fatigability ≥10**	87 (47.5)	62 (37.6)	48 (49.0)	0.09

Physical activity was measured using a 7-day free-living accelerometer worn on the right mid-thigh; n=149 and n=59 wore the devices and had valid data at 6 Month and 18 Month. MVPA = moderate-to-vigorous physical activity

Peak oxygen consumption (VO_2peak) was measured using a maximal cardiopulmonary treadmill exercise test, n=158 and n=77 completed the test and had a valid VO_2peak at 6 Month and 18 Month visits.

Cost-capacity ratio was calculated as the ratio of average oxygen consumption from a 5-minute, 0% grade treadmill walk at 2.0 MPH relative to peak oxygen consumption. Scores range from 0 to 100%.

Pittsburgh Fatigability Scale (PFS) Physical score was measured using the validated 10-item questionnaire. Scores range from 0 to 50 and a score ≥15 indicates more severe fatigability. N=168 and N=137 completed the PFS at 6 Month and 18 Month visits.

Borg RPE Fatigability was measured immediately at the end of a 5-minute, 0% grade treadmill walk at 2.0 MPH. Scores range from 6 to 20 and a score ≥10 indicates high fatigability. N=159 and N=79 completed the test at 6 Month and 18 Month.

Table S2. Comparison of Baseline Characteristics between those who met Weight Loss Goal of at least 7% body weight reduction vs those who did not meet weight loss goal, N=168

	Total<br>N=168	Did Meet WL Goal<br>n=102	Did not Meet WL Goal<br>n=66	p-trend
**Age, years**	69.4±4.2	69.3±3.4	69.8±5.3	0.47
**Female**	128 (76.2)	76 (59.4)	52 (40.6)	0.52
**White non-Hispanic**	124 (73.8)	82 (66.1)	42 (33.9)	**0.03**
**Education**				0.93
**High school**	19 (11.3)	11 (57.9)	8 (42.1)	
**College**	97 (57.8)	60 (61.9)	37 (38.1)	
**Postgraduate**	52 (31.0)	31 (59.6)	21 (40.4)	
**Height, m**	1.6±0.1	1.6±0.1	1.6±0.1	0.32
**Weight, kg**	96.4±14.1	97.4±15.1	95.1±12.4	0.31
**Body mass index, kg/m^2**	35.6±3.8	35.7±4.0	35.5±3.4	0.69
**Step count, /d**	6388±2439	6394±2444	6378±2455	0.97
**MVPA, min/d**	9.0±8.6	9.9±9.4	7.9±7.3	0.21
**PFS Physical score, 0-50**	20.7±7.4	20.4±7.9	21.3±6.8	0.43
**PFS Physical score ≥15**	135 (80.3)	77 (57.0)	58 (43.0)	**0.04**
**RPE Fatigability, 6-20**	9.6±1.9	9.5±1.9	9.8±2.1	0.42
**RPE Fatigability ≥10**	79 (47.0)	46 (58.2)	33 (41.8)	0.45

N=168 includes participants who had available weight data at baseline and at 6 months. N=15 had missing weight data at 6 months

Note: No differences by intervention group (p=0.96) or recruitment wave (p=0.66)

MVPA = moderate-vigorous physical activity

PFS = Pittsburgh Fatigability Scale

RPE = Rating of Perceived Exertion
